# Genetics of randomly bred cats support the cradle of cat domestication being in the Near East

**DOI:** 10.1038/s41437-022-00568-4

**Published:** 2022-11-01

**Authors:** Sara M. Nilson, Barbara Gandolfi, Robert A. Grahn, Jennifer D. Kurushima, Monika J. Lipinski, Ettore Randi, Nashwa E. Waly, Carlos Driscoll, Hugo Murua Escobar, Rolf K. Schuster, Soichi Maruyama, Norma Labarthe, Bruno B. Chomel, Sankar Kumar Ghosh, Haydar Ozpinar, Hyung-Chul Rah, Javier Millán, Flavya Mendes-de-Almeida, Julie K. Levy, Elke Heitz, Margie A. Scherk, Paulo C. Alves, Jared E. Decker, Leslie A. Lyons

**Affiliations:** 1 Division of Animal Sciences, University of Missouri, Columbia, MO 65211 USA; 2 Department of Population Health and Reproduction, School of Veterinary Medicine, University of California, Davis, CA 95616 USA; 3 Department of Chemistry and Bioscience, Aalborg University, Fredrik Bajers Vej 7H, 9220 Aalborg Øst, Denmark; 4 Department of Animal Medicine, Faculty of Veterinary Medicine, Assuit University, 71526 Assiut, Egypt; 5 Galton Corporation, Frederick, MD 21701 USA; 6 Clinic for Hematology, Oncology and Palliative Care, University Medical Center Rostock, 18055 Rostock, Germany; 7 Central Veterinary Research Laboratory, Dubai, United Arab Emirates; 8 Laboratory of Veterinary Public Health, Nihon University, 1866 Kameino, Fujisawa, Kanagawa 252-0880 Japan; 9 Programa de Bioética, Ética Aplicada e Saúde Coletiva, Fundação Oswaldo Cruz, Rio de Janeiro, RJ 21040-360 Brazil; 10 Programa de Pós-Graduação em Medicina Veterinária – Clínica e Reprodução Animal, Faculdade de Veterinária, Universidade Federal Fluminense, Rua Vital Brazil Filho 64, Niterói, RJ 24230-340 Brazil; 11 Department of Biotechnology, Assam University, Silchar, 788011 India; 12 Graduate School of Health Sciences, Istanbul Gedik University, 34876 İstanbul, Turkey; 13 Research Institute of Veterinary Medicine, College of Veterinary Medicine, Chungbuk National University, Cheongju, 28644 South Korea; 14 Instituto Agroalimentario de Aragón-IA2 (Universidad de Zaragoza-CITA), Miguel Servet 177, 50013 Zaragoza, Spain; 15 Fundación ARAID, Avda. de Ranillas, 50018 Zaragoza, Spain; 16 Facultad de Ciencias de la Vida, Universidad Andres Bello, Santiago, Chile; 17 Maddie’s Shelter Medicine Program, College of Veterinary Medicine, University of Florida, Gainesville, FL 32608 USA; 18 Al Qurum Vet Clinic, Muscat 118, Oman; 19 CatsINK, Vancouver, BC V5N 4Z4 Canada; 20 CIBIO/InBIO, Centro de Investigação em Biodiversidade e Recursos Genéticos/InBIO Associate Lab & Faculdade de Ciências, Universidade do Porto, Campus e Vairão, 4485–661 Vila do Conde, Portugal; 21 Wildlife Biology Program, University of Montana, Missoula, MT 59812 USA; 22 Institute for Data Science and Informatics, University of Missouri, Columbia, MO 65211 USA; 23 Department of Veterinary Medicine & Surgery, College of Veterinary Medicine, University of Missouri, Columbia, MO 65211 USA

**Keywords:** Genetic variation, Molecular evolution

## Abstract

Cat domestication likely initiated as a symbiotic relationship between wildcats (*Felis silvestris* subspecies) and the peoples of developing agrarian societies in the Fertile Crescent. As humans transitioned from hunter-gatherers to farmers ~12,000 years ago, bold wildcats likely capitalized on increased prey density (i.e., rodents). Humans benefited from the cats’ predation on these vermin. To refine the site(s) of cat domestication, over 1000 random-bred cats of primarily Eurasian descent were genotyped for single-nucleotide variants and short tandem repeats. The overall cat population structure suggested a single worldwide population with significant isolation by the distance of peripheral subpopulations. The cat population heterozygosity decreased as genetic distance from the proposed cat progenitor’s (*F.s. lybica*) natural habitat increased. Domestic cat origins are focused in the eastern Mediterranean Basin, spreading to nearby islands, and southernly via the Levantine coast into the Nile Valley. Cat population diversity supports the migration patterns of humans and other symbiotic species.

## Introduction

The domestication and the geographical origins of the household cat (*Felis silvestris catus; Felis catus* (Kitchener et al. 2017)) have been partially reconstructed from archaeological discoveries, cultural and artistic depictions, and genetics evaluations of ancient and modern felids (Vigne et al. 2004; Driscoll et al. 2007; Faure and Kitchener 2009; Zeder 2012; Ottoni et al. 2017; Cucchi et al. 2020). The cat’s domestication process likely initiated ~12,000 years ago in the Fertile Crescent with the initial contact between *Felis silvestris lybica* and farmers. The advent of agriculture altered human culture from nomadic hunter-gatherers to more sedentary lifestyles, leading to the establishment of increasingly larger settlements. Grain stores and refuse from developing societies attracted mice, which led to a synanthropic trinity between humans, rodents, and felids. Archeological evidence suggests the domestication process of *F.s. lybica* individuals initiated in the Near East with agrarian societal development within the Fertile Crescent and the Levant, intensified in Egypt along with cultural worships, leading to human migration and trade facilitating the domesticated feline dispora (Vigne et al. 2004; Faure and Kitchener 2009; Van Neer et al. 2014; Ottoni et al. 2017). Feline remains, buried alongside human remains, were discovered at an archeological site dating to ~9500 years ago, suggesting humans had formed a relationship with cats and transported cats to Cyprus (Vigne et al. 2004, 2012). The earliest remains of suggested tamed cats in Egypt date to the fourth millennium BC (Baldwin 1975; Málek 2006; Van Neer et al. 2014) and suggest felines became integral to Egyptian culture, culminating in thousands of mummified cats as votive offerings (Baldwin 1975; Faure and Kitchener 2009; Ikram and Hawass 2004; Kurushima et al. 2012). Beginning in the first millennium BC, progeny of the Egyptian tamed cats were spread through trade and maritime routes by Phoenician, Carthaginian, Greek, Etruscan “cat-thief” traders and later by the Romans (Baldwin 1975; Faure and Kitchener 2009; Ottoni et al. 2017).

To genetically assess wildcats, feral domestic, and fancy-breed domestic cat relationships, a phylogenetic study was conducted with mitochondrial DNA (mtDNA) sequences of 2604 base pairs from *ND5* and *ND6*, and 36 short tandem repeats (STR) genotypes (Driscoll et al. 2007). A singular domestication origin in the Near East, arising from *F.s. lybica* was suggested; however, a limited sampling of wildcat subspecies was available. An expanded study of random-bred, domestic breeds, and wildcats with STR data suggested the most likely origin of domestication was the Mediterranean Basin. In addition, four significant genetic distinctions were identified amongst 13 Eurasia cat populations (Lipinski et al. 2008) based on allele frequencies and Bayesian clustering, particularly for the Far Eastern, Mediterranean, Western European, and Kenyan cats. Studies of the mtDNA control region variation in random-bred cats have also supported four to five major cat lineages with 12 common mitotypes representing maternal lineage diversity (Grahn et al. 2011). An mtDNA study of mainly ancient and some modern felid samples from Europe, Africa, and Asia also traced modern felines to multiple *F.s. lybica* lineages within the Fertile Crescent (Ottoni et al. 2017). The first occurrence of the mitochondrial haplotype A* of a *F.s. lybica/catus* species was reported in Bulgaria ~6400 years ago that is prior to the occurrence in Poland about 3400–2500 years ago, thereby, extending *F.s. lybica/catus* into a shared niche with *F.s. silvestris* from Anatolia to Eastern Europe (Krajcarz et al. 2016, 2020; Ottoni et al. 2017; Baca et al. 2018).

Cat domestication is likely commensal with agricultural development. With the onset of the Holocene ~10,000 years ago and differences in regional climate changes, agriculture developed independently in several different global regions: the Near East likely the earliest, followed closely by agricultural sites in China, Southeast Asia, and later in the Americas (Bellwood et al. 2005). Archeological discoveries of human remains and artifacts in the Near East and the middle Yangtze and Yellow Rivers in China indicate the earliest emergence of complex civilizations (Baldwin 1975; Bar-Yosef 1998; Hu et al. 2014). The Indus Valley of modern-day Pakistan is also argued as a historical center for agricultural development (Bellwood et al. 2005). Recent studies of Chinese random-bred cats and the local wildcat species/subspecies (*F.s. bieti*) suggest the noted introgression of this wildcat with random-bred cats in China does not explain the distinctive genetics of Far Eastern and Western European random-bred cats; further, the agricultural center near the middle Yangtze and Yellow Rivers is likely not a second domestication site for cats (Yu et al. 2021). While these previous studies all support the domesticated *F.s. catus* arose from *F.s. lybica* originating in the Near East, cats from other Eurasian regions of early agricultural development, including within other Near and Middle Eastern regions as well as the Indus Valley of Pakistan, have not been examined in the context of contributing to feline domestication, which may account for the significant genetic distinction between Eastern and Western cat populations found in additional studies (Lipinski et al. 2008). Although European colonization occurred a few hundred years ago, regional cat populations tend to represent the initial domesticates of colonization and not unique or highly admixed populations, such as the cats in Australia (Lipinski et al. 2008; Koch et al. 2015; Spencer et al. 2016) as well as, North America and Nairobi, Kenya that are both genetically most similar to cats of Western Europe (Lipinski et al. 2008). Interestingly, cats of Madagascar suggest a genetic similarity with cats from the Arabia Sea trade routes, namely the Kenyan islands of Lamu and Pate, Oman, Kuwait, and Iran, and not cats imported by more recent colonists from France (Sauther et al. 2020), further suggesting demographic stasis and the original influx of cats to a region may have the strongest influence on genetic signatures, rather than more recent migrants. Ancient DNA studies often suggest the converse; modern populations have no power to infer the dynamics of temporal populations movements (see reviews, Freedman and Wayne 2017; Frantz et al. 2020).

Since cats have and continue to perform their key role of vermin control without human assistance, random-bred cats may have escaped intense selective pressures due to breed formation, such as the strong selection pressure for particular phenotypes (Kurushima et al. 2013). As such, random-bred cats represent an intermediate step in cat domestication, between wildcats and highly selected cat breeds. While modern populations only represent the latest epoch of migration and admixture (Pickrell and Reich 2014), random-bred cats likely represent clearer patterns of historical diversity than fancy-breed cats. The historical time period reflected by random-bred cat genetic diversity is unknown and likely variable.

This study investigated the genetic diversity of modern cat populations to determine if current genetic distinctions are discrete, suggesting possible secondary genetic progenitors, or a continuum of diversity from a population center and due to isolation by distance. To clarify historical cat population dynamics, population sampling of random-bred cats was focused near regions of early human agricultural developments, with extensive representation from the Near/Middle East, Pakistan, and near the Yellow River in China, with the addition of populations across Eurasia, from Southeast Asia to Great Britain. The geographical origins of cat domestication should be near the centers of cat genetic diversity.

## Materials and methods

### Sample collection

Samples were collected via buccal (cheek) swabs, Flinders Technology Associates (FTA) Cards (Whatman International Ltd., Maidstone, UK), gonads from neuter clinics, and donated EDTA whole blood samples. DNA isolations were conducted following the manufacturer’s protocol using the method appropriate for the sample, including QIAamp DNA blood mini kits, Qiagen DNA Easy kits (Qiagen, Valencia, CA, USA), organic extractions or methods for FTA card blood spots (Gandolfi et al. 2016). Samples were amplified using whole genome amplification (REPLI-g Mini Kit, Qiagen) when DNA quantity was insufficient.

Cat samples (*n* = 564) from a previous study included random-bred cats from 17 locations (Supplementary Tables 1 and 2) (Lipinski et al. 2008; Kurushima 2011). Cats previously labeled in the previous study as from Singapore were actually from Taiwan. Four African wildcat samples (*F.s. lybica*) were collected as part of other studies from Western Sahara, Morocco, Tunisia, and Mauritania, and provided as extracted and whole genome amplified DNA (Randi et al. 2001; Lecis et al. 2006; Oliveira et al. 2015). The STR data for the cats from Madagascar (*n* = 27) has been previously published (Sauther et al. 2020). Domestic cat samples from Portugal and Italy have been previously analyzed, but new data were generated for the Madagascar study to ensure allele size binning accuracy (Lecis et al. 2006; Oliveira et al. 2008a, 2008b). Additional random-bred cat samples were collected from 30 new countries and additional population locations within several countries including Brazil, China, Egypt, Italy, Kenya, South Korea, and the USA (Supplementary Tables 1 and 2). For the STR analyses, 1857 random-bred cats and the four African wildcats were genotyped. For the single-nucleotide polymorphism (SNP) analyses, 969 random-bred cats were genotyped. In addition, the same four African wildcats and 10 cats collected as roadkill from Spain, putative wildcat hybrids, were included in the SNP dataset (Oliveira et al. 2008a, 2015).

### Genotyping

Thirty-six autosomal STRs were genotyped following the PCR and analysis procedures in a previous study (Lipinski et al. 2008) (Supplementary Table 3). Unlinked non-coding autosomal SNPs (*n* = 132) were selected to represent all autosomes from the 1.9× coverage cat genomic sequence, which were defined by one Abyssinian cat (Pontius et al. 2007). The SNPs have been remapped to cat genome assembly Felis Catus 9.0 (Buckley et al. 2020). Primers were designed with the VeraCode Assay Designer software (Illumina Inc., San Diego, CA, USA). The SNPs had a Ranking Score of 0.75 or higher (with a mean design score of 0.95) and a Gen Train Score of >0.55 (Supplementary Table 4). Golden Gate Assay amplification and BeadXpress reads were performed per the manufacturer’s protocol (Illumina Inc., Foster City, CA) on 50–500 ng of DNA or whole genome amplified product. BeadStudio software v. 3.1.3.0 with the Genotyping module v. 3.2.23 (Illumina Inc.) was used to analyze the data. In PLINK v1.9, quality control for minor allele frequency was set at 0.005, and genotype call rate was set at 0.8 (Chang et al. 2015). The genotyping data for the project are presented in Supplementary Files 1 and 2.

### Principal component analysis

To project the genetic similarities among individuals, principal component analysis (PCA) was performed for the SNP data with the smartpca program from the EIGENSOFT package (Patterson et al. 2006). To determine the potential effect of population size, four different PCAs were generated by grouping individuals by (1) sample location, (2) country, (3) sample location with populations randomly reduced to a maximum of 25 individuals, and (4) country with populations randomly reduced to a maximum of 40 individuals. Due to minimal visual differences, all further SNP analyses were conducted on the country grouped data with populations randomly reduced to a maximum of 40 individuals per country, totaling 969 random-bred felines in the dataset. A PCA of the STR dataset was conducted with the R package adegenet v2.1.1 (Jombart 2008).

### Population structure

A variational Bayesian framework, fastSTRUCTURE, estimates the admixture proportions of individuals when given *K* populations (Raj et al. 2014). The fastSTRUCTURE software proposes two metrics to select and identify *K*: the *K* that maximizes the log-marginal likelihood lower bound of the dataset ($K_\varepsilon ^ \ast$) and the minimum *K* that accounts for a cumulative ancestry of 99.99% ($K_{ \oslash C}$). The analyses using fastSTRUCTURE were run independently for a *K* of 1–20 for the SNP data. As fastSTRUCTURE is specific to biallelic data, a Bayesian clustering method, STRUCTURE v2.3.4, was utilized for STR analyses for jointly inferring the *K* populations represented and probabilistically assigning each individual to one or more populations (Pritchard et al. 2000). Overall, STRUCTURE was run from a *K* of 1–35 with each independent *K* run 20 times. Runs consisted of a 50,000 burn-in period with 50,000 MCMC replications, and the results were averaged with CLUMPP v1.1.2 (Jakobsson and Rosenberg 2007). Averaged results were calculated only for *K* of 1–5 as higher *K* values did not converge, most likely due to little population structure differences. The ∆*K* distribution was calculated following the process implemented by Evanno et al. (2005) to determine an optimal value of *K*.

### Admixture

To identify admixture and support fastSTRUCTURE and STRUCTURE observations, *f*_*3*_ statistics were calculated among all sample location populations (significant results presented in Supplementary Table 5) for the SNP dataset, excluding small populations with less than five individuals and those from the Americas and Australia, with the *threepop* component of the TreeMix program (Pickrell and Pritchard 2012).

### Isolation by distance

To formally test for isolation by distance at the finest geographical scale, SNP populations were reclassified back to their sample location labels to achieve fine-scale results (Supplementary Tables 6 and 7). Due to potential bias, sample locations with less than five individuals were removed from further analyses. In addition, sample locations from the Americas and Australia were excluded due to strong evidence supporting European ancestry and geographic distance being exaggerated due to human-mediated migration. The remaining sample location populations had *f*_*3*_ statistics calculated and those populations with significant values were removed to reduce noise generated by admixture possibly due to migration events (see Admixture). Removal of admixed locations was done to strengthen the relationship between modern samples and ancient processes by removing more recent admixture events. For the SNP data, 24 sample location populations were analyzed, not including the *F.s. lybica* and the wildcat hybrid populations. For the STR data, the same individuals from the SNP dataset were used resulting in 22 populations. The two populations lost due to no STR genotypes were from Spain and Portugal. Isolation by distance was tested with a Mantel test between calculated matrices of geographical distances (geodesic in meters from latitude and longitude coordinates) and Cavalli-Sforza and Edwards chord genetic distances with the adegenet and geodist R packages (Jombart 2008; Karney 2013; Séré et al. 2017). Cavalli-Sforza and Edwards chord genetic distances were used as it was previously shown to be a more powerful approach for isolation by distance (Séré et al. 2017). The Mantel test results are calculated with a Monte-Carlo test with 999 replicates; the final reported correlation and *p* value are the average of 1000 independent Monte-Carlo tests. To further explore expansion and migration patterns, isolation by distance was calculated among all of the samples collected in the contiguous United States of America for both data types.

### Genetic diversity

Observed and expected heterozygosities were calculated for the SNP and STR populations used in the isolation by distance analyses, and for all sample locations with the adegenet R package (Jombart 2008). F-statistics were calculated for the SNP and STR random-bred cat data on a worldwide population level with the hierfstat R package (Goudet 2005). In addition, F_IS_ statistics were calculated for all sample locations with the equation: F_IS_ = 1 – (*H*_obs_/*H*_exp_) (Supplementary Table 8).

## Results

The genotyped cat samples consisted of 1987 random-bred cats (*F.s. catus*), four African wildcats (*F.s. lybica*), and 10 putative hybrids of domestic and European wildcats (*F.s. silvestris*) (Oliveira et al. 2015). Random-bred cats (*n* = 839) genotyped for both SNPs and STRs, 1018 cats were genotyped for STRs only and 130 cats were genotyped for SNPs only. The four African wildcats were genotyped for both SNPs and STRs, while the 10 putative hybrids of domestic and European wildcats were only genotyped for the SNPs. The random-bred cats represent over 40 countries including over 85 sampling sites (Supplementary Tables 1 and 2). The distribution of the populations and marker types is depicted in Supplementary Fig. 1. A majority of sampling was focused on the European and Asian continents, particularly the Near East region.

**Fig. 1:**
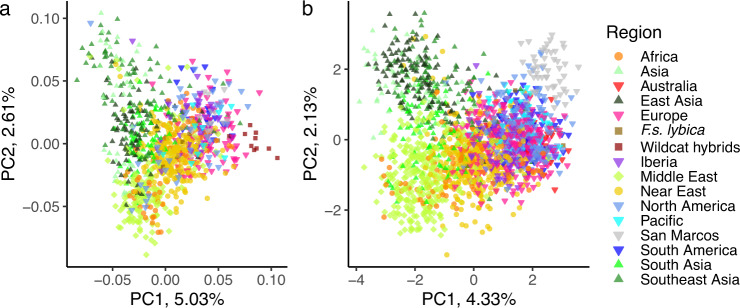
Principal component analyses (PCA) of genetic variation in random-bred and wildcat felines. **a** PCA plot of SNP data (*N* = 983). **b** PCA plot of STR data (*N* = 1861). A single point represents an individual, the shape represents a geographic region, the color represents a geographic sub-region. The two wildcat populations are denoted by squares of different colors. Middle Eastern, South Asia, and Western European cats form the peripheral subpopulations of random-bred cats. The wildcat hybrids and the island population of San Marcos, Baja California, are additional peripheral populations for **a** and **b**, respectively.

The PCA of the SNP and STR data sets have similar patterns (Fig. 1). Principal component 1 (PC1) forms a cline of felines from Asia and the Middle East (negative values) to Europe and the Americas (positive values) with felines from Africa and the Near East central to the peripheral populations. Principal component 2 (PC2) highlights differences between Asian cats from the Near East (Cyprus, Israel, Egypt, Jordan, Lebanon, Greece), the Middle East (Bahrain, Iran, Iraq, Kuwait, Oman, Pakistan, UAE), and African cats (Tunisia, Kenya, Madagascar). In both data sets, the four African wildcats (*F.s. lybica*), which are considered the progenitor subspecies for the domestic cat, are positioned mainly with the felines from the Near East, near the center of the PCA space. However, the putative wildcat hybrids in the SNP dataset cluster peripherally from the random-bred felines, and appear more closely related to the felines from Western Europe, including cats from the Americas, which could be due to introgression among the populations.

The San Marcos Island (Baja California Sur, Mexico) population in the STR dataset diverges from the European and American felines, reflective of a small, isolated island population. Both PCA reflect genetic divergence due to geographic separation, but the populations form a cline rather than clear geographical clusters. Southeastern and East Asian cats are located at one periphery of the distribution, as are the Near Eastern and Mediterranean cats in another, with the Western European cats in the third. Only 8–9% of the total genetic variability could be attributed to differences among the cat populations (STR F_ST_ = 0.078; SNP F_ST_ = 0.088). On average, the local populations had a deficit of heterozygotes of 6–9% (STR F_IS_ = 0.088; SNP F_IS_ = 0.063) whereas the total worldwide random-bred population had a deficit of heterozygotes of 15–16% (STR F_IT_ = 0.159; SNP F_IT_ = 0.146).

**Fig. 2:**
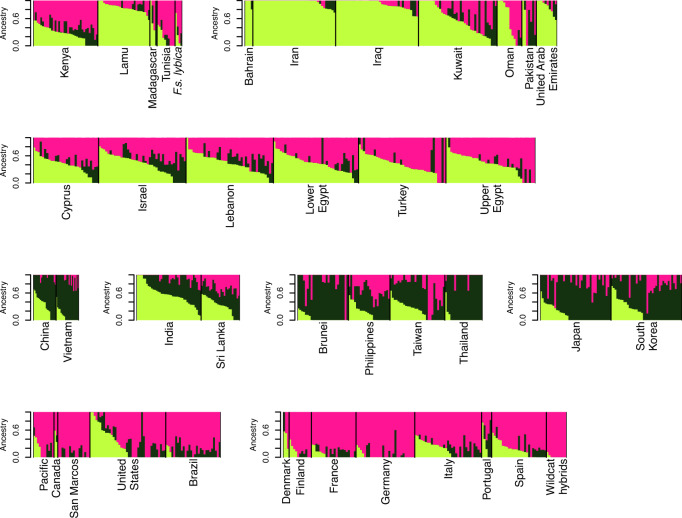
Random-bred cat population SNP fastSTRUCTURE plot of *K* = 3. Population contributions are represented by different colors, individual vertical bars represent an individual, and populations are separated by black lines.

Population structure was estimated across both data sets to gain insight into the admixture of the current random-bred felines. For the SNP data, a *K* of 1 explains 99.99% of the variation in the dataset ($K_{ \oslash C}$ statistic, (Raj et al. 2014)) suggesting the worldwide random-bred feline populations do not form genetically distinct clusters, even though the cats have been geographically separated. A *K* of 2 maximizes the log-marginal likelihood lower bound ($K_\varepsilon ^ \ast$ statistic, (Raj et al. 2014)) separating felines between Western European ancestry, and Asian/Middle Eastern/Mediterranean ancestry (Supplementary Fig. 2). Felines from Africa (Nairobi, Kenya, and Tunis, Tunisia) and Western Europe share genetic similarities between the two ancestry assignments. Cats in the Americas have a genetic profile typical of Western European cats. The African cats from the eastern islands of Kenya, Lamu and Pate, share genetic similarities with cats from the Middle East and the Eastern Mediterranean. These similarities are maintained through higher levels of sub-structure. The *K* of 2 ancestry pattern reflects population positionings in the PCA along PC1. As the *K* increases to 3 and 4, the Asian felines reflect PC2, and the sub-regional distinction appears with East Asia and Southeast Asia (Figs. 1 and 2 and Supplementary Fig. 3). As *K* increases up to 5, the island population of San Marcos appears distinct, with additional sub-regional assignments within Western Europe, Mediterranean, Near/Middle East, and East Asia (Supplementary Fig. 4). Countries such as India and Sri Lanka appear to be highly admixed. The four African wildcats are similar to a typical Western European population and the putative hybrids of domestic and European wildcats are a more cohesive grouping in which Western European cats share ancestry.

**Fig. 3:**
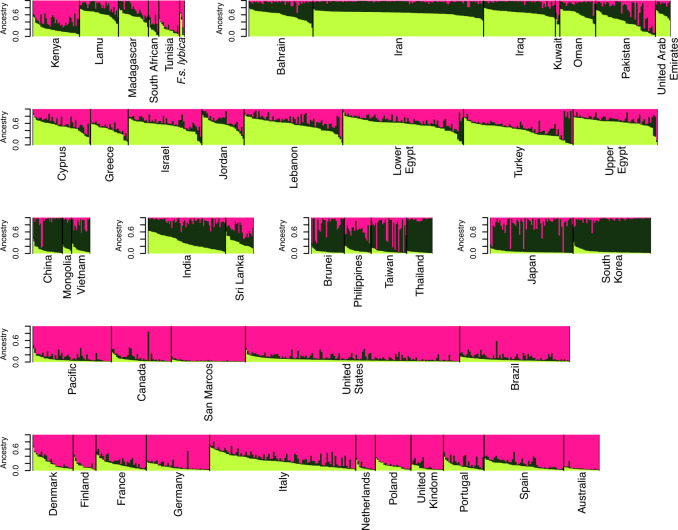
Random-bred cat population STR STRUCTURE plot of *K* = 3. Population contributions are represented by different colors, individual vertical bars represent an individual, and populations are separated by black lines.

Similar population structuring is depicted by the STR analyses. For the STRs, the modal value of the ∆*K* distribution was at *K* = 2. The ancestral populations were split between Western Europe versus Middle East/Asia, which is consistent with the SNP data (Supplementary Fig. 5). Random-bred cats from Africa (South Africa, Nairobi, Kenya, and Tunis, Tunisia) and the Near East were mixed almost equally between these two ancestry assignments. As *K* increases, more geographical separation is depicted: *K* of 3 distinguishes Asian cats from the Mediterranean/Near/Middle Eastern cats, a *K* of 4 separates the Mediterranean/Near East cats from Middle East felids, and a *K* of 5 brings out the island population from San Marcos (Fig. 3 and Supplementary Figs. 6 and 7). Overall, the population structure between the SNPs and STRs is concordant and consistent with the patterns observed in the PCA (Engelhardt and Stephens 2010), supporting the inference that the worldwide random-bred subpopulations are a single population with genetic differentiation due to separation by geographic distance. The most observable difference between SNPs and STRs is the SNPs differentiate Southeast and Eastern Asian cats at *K* = 4 while STRs maintain the Asian cats as a stronger cluster and differentiate Mediterranean/Near Eastern cats from the cats of the Middle East (Supplementary Figs. 3 and 6).

The allele frequencies of the cat populations were analyzed to calculate *f*_*3*_ statistics with corresponding *z*-scores to evaluate possible admixture (Supplementary Table 5) (Reich et al. 2009). There are 234 of 51,888 comparisons with a *z*-score ≤ −2 (0.45%), supporting admixture within the 22 target populations. The sample population from Lahore, Pakistan has the lowest *z*-score of −4.9 and had 56 significant *z*-scores with other populations that are highly indicative of admixture. Populations most frequently contributing to significant admixture as parent (i.e., donor) populations include Thailand, Vietnam, the wildcat hybrids, and Asyut, Egypt.

**Fig. 4:**
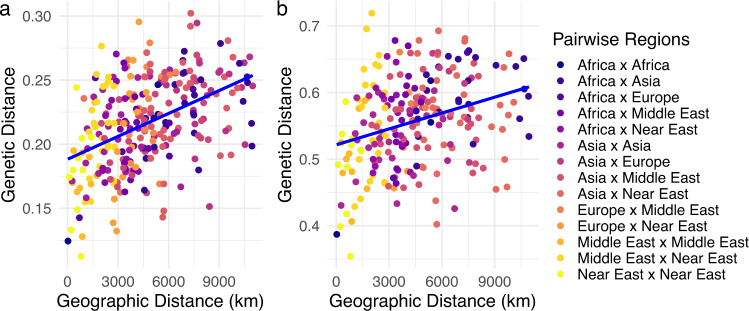
Comparison of geographic distance and genetic distance of random-bred cat populations. **a** Plot of SNP data with 24 sample locations with a regression line indicating a correlation of 0.447 with a *p* value of 0.001. **b** Plot of STR data with 22 sample locations with a regression line indicating a correlation of 0.302 with a *p* value of 0.0076. Each point represents an individual pairwise comparison of sample location populations.

Since population structure analyses suggest a single population with possible differentiation due to geographic separation, isolation by distance was formally tested among the sample location populations in Europe, Africa, Near East, Middle East, and Asia for which evidence of admixture was not observed from *f*_*3*_ statistics (see Admixture and Supplementary Table 5). When the population pairwise geographic distances are plotted against the Cavalli-Sforza and Edwards chord genetic distances (Séré et al. 2017), a clear trend is observed; as the geographic distance increases between populations, the genetic distance also increases (Fig. 4 and Supplementary Tables 6 and 7). The Mantel test between distance matrices resulted in a positive correlation of 0.447 with a *p* value of 0.001 for the SNP data, and a positive correlation of 0.302 with a *p* value of 0.0076 for the STR data. When the admixed populations were included in a separate Mantel test for isolation by distance, the SNP data had a positive correlation of 0.369 with a *p* value of 0.001, and the STR data had a positive correlation of 0.23 with a *p* value of 0.0025. The ~10% decrease in correlations between genetic and geographic distance when the admixed populations were included could be due to the increased genetic noise from migrants. Conversely, isolation by distance analyses were not significant (SNP *p* value = 0.871; STR *p* value = 0.405) for random-bred cats in the contiguous United States of America, suggesting multiple importations of felines into the USA and little geographical structure in the genomic data.

**Fig. 5:**
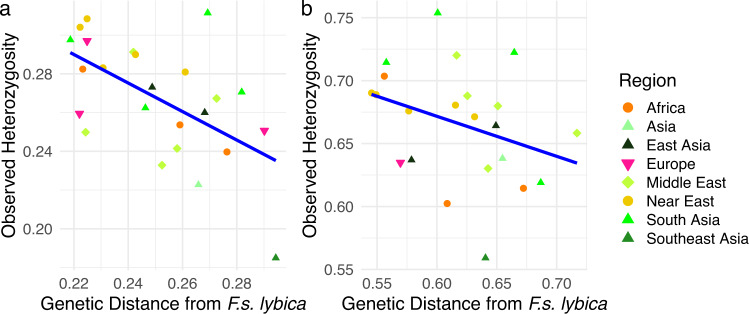
Comparison of genetic distance from *F.s. lybica* and observed heterozygosity. **a** Plot of SNP data with a regression line indicating a correlation of −0.57 with a *p* value of 0.0034. **b** Plot of STR data with a regression line indicating a correlation of −0.33 with a *p* value of 0.13. Each point represents a sample location population, shape represents a geographical region, and color represents a geographical sub-region.

**Fig. 6:**
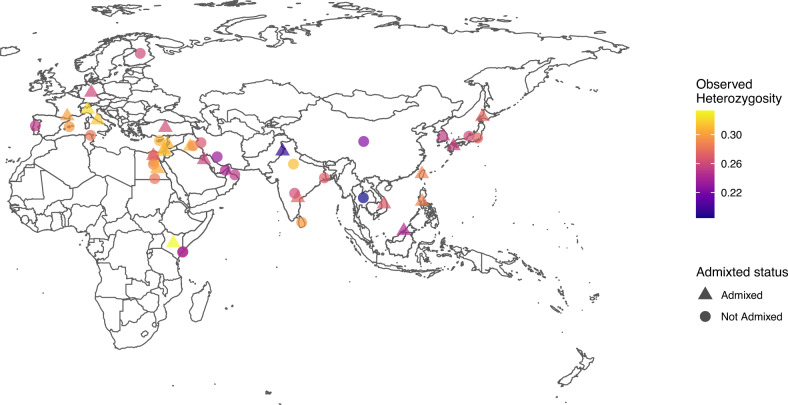
Observed heterozygosity by sample location of SNP data for random-bred cat populations of Eurasia. Each point represents a sample location population with the color showing the calculated observed heterozygosity. The triangle shape indicates an admixed population with a significant *f*_*3*_ statistic, and the circle shape represents non-admixed populations. Populations of yellow and light orange shades are focused in the Near East and Mediterranean Basin.

Based on the significant isolation by distance, observed and expected heterozygosities were calculated for each sample site. When the observed heterozygosity is plotted against the genetic distance from the domestic progenitor, *F.s. lybica*, a negative relationship is identified; as the genetic distance from *F.s. lybica* increases the observed heterozygosity decreases (Fig. 5). There is a negative correlation for the SNP data of −0.57 with a *p* value of 0.0034 while the STR correlation is −0.33 with a *p* value of 0.13. To explore the geographic and observed heterozygosity relationship further, the populations were plotted on a map to identify an epicenter of high diversity that decreases outwards in a radial fashion as expected from a center of domestication (Fig. 6 and Supplementary Table 8). The centers of diversity are focused on the Mediterranean side of the Fertile Crescent, including the Levant, and expanding into the Nile Valley and Mesopotamia. Cat populations with high heterozygosity are also identified in Agra, India, Sri Lanka, and the island population of Majorca, Spain (Supplementary Table 8).

## Discussion

Throughout the world, the domestic cat is a beloved and charismatic companion animal. Although as popular of a pet as the domestic dog, the origins of the domestic cat are less studied. Random-bred cats (i.e., feral, moggie, alley, house, community, street, or barn cats) remain a behaviorally semi-domesticated species that can quickly revert to a wild state. While they have a low survival rate in the wild, their high reproductive capacity increases population size (Nutter et al. 2004). As apex predators, this reversion capability has often been exploited to eradicate invasive animals from island populations, whereas later, the cats themselves became invasive alien species (Rendall et al. 2021; Plein et al. 2022).

Here, the random-bred cats of the study represent semi-domesticated animals that lie somewhere between “habituation” and “commercial breeds and pets” on the commensal domestication trajectory (Zeder 2012; Larson and Burger 2013). For cats, human assistance is not necessarily required for mating, shelter, safety, or the procurement of food (Driscoll et al. 2009). The cat’s semi-domesticated behavioral state is consistent with weaker human-influenced artificial selection pressures on the species. Although cats may have been domesticated at approximately the same time as many agricultural species, ~8000–10,000 years ago, cats have scavenged refuge piles and curbed vermin populations during their symbiotic relationship with humans (Clutton-Brock 1988). Therefore, for the past several thousand years, cats have not been transformed drastically in form or function, unlike dogs and economically important species. Only for the past ~200 years, cat breeds, not random-bred cats, have been selected for mainly monogenic aesthetic traits undergoing novelty selection on a small number of loci and likely a small portion of the genome. Minor structural differences and no functional behavioral differences were present in cats when the first cat show took place in 1871 (The Cat-Show 1871). The semi-domesticated nature of random-bred cats makes them an excellent resource to understand cat population origins, domestication, and dispersal.

SNP and STR genotypes of an extended and fine-scale sampling of Eurasian cats demonstrated domestication most likely occurred in the concentrated region of the Fertile Crescent. The focused sampling plan was to test alternative hypotheses of multiple domestication centers in (1) Near East, (2) China, and (3) Southeast Asia against the null hypothesis of a single domestication center. This focused sampling could also identify distinct populations indicative of admixture with wild relatives. However, despite this intensive sampling, only the Near East is suggested as a site of cat domestication indicating a pattern of dispersal outwards from regions like the Levant and the Nile Valley, while elsewhere in the world lacks this pattern (Vigne et al. 2004; Driscoll et al. 2007; Lipinski et al. 2008). For other domesticated species, isolation by distance testing and genetic diversity measurements reveal a pattern of expansion from the domesticated founders (Ramachandran et al. 2005; Scheu et al. 2015; Malomane et al. 2021).

Previous genetic studies examined the extremes of the geographical locations while the current research included bridging populations, which revealed the structure of worldwide random-bred populations is nearly a panmictic population with evidence of isolation by distance at the peripheries of their migration (Lipinski et al. 2008). As found for human populations (Barbujani et al. 1997), a majority of genetic diversity is explained within populations, and distinctions can be observed only at the peripheries of migration patterns and do not account for the vast genetic diversity of cats. This pattern of isolation by distance, with the highest levels of diversity near sites of domestication, is observed in other species. Chickens, like cats, dispersed from a domestication center by human-mediated migration, and the majority of genetic diversity variation is explained by genetic distance to the wild populations (Malomane et al. 2021). Village dogs, like random-bred cats, are considered to be free-breeding with minimal admixture due to isolation and have escaped human-mediated inbreeding (Shannon et al. 2015). Although the location of dog domestication is disputed (Bergström et al. 2020), genetic signatures have been used to infer a Central Asia domestication of dogs. Patterns of short-range linkage disequilibrium decay were found to be lowest in village dog populations from Central Asia with rates rising as geographical distance increased (Shannon et al. 2015). After filtering admixed populations to remove the most recent epoch of admixture and improve the fit between modern samples and ancient ancestry patterns, the random-bred cat results suggest a similar pattern: genetic diversity is higher in populations located where the progenitor species began to interact with humans resulting in a shorter genetic distance and heterozygosity decreasing as geographic distance increases out from this origin. Studies using ancient DNA of domesticated cats may reveal a more complicated process (MacHugh et al. 2017), but the pattern revealed in random-bred cats is striking and agrees with archeological evidence. Unlike many domestication studies that must use modern breeds for comparisons, these random-bred cats have likely had less selection, weaker founder effects, and lower genetic loss by drift since cats are under fewer constraints by humans.

The cat diaspora is relatively more recent than for humans or canines. As European maritime exploration to conquer and settle new lands increased, felines were brought on ships for trade and to safeguard food and wares from rodents (Faure and Kitchener 2009). Migration of cats rose with imperialism exploration and colonization, which increased the number of ships traveling to the Americas (Todd 1977). The data suggest cats in distant areas from the Near East, including Australia, the Americas, and colonial regions such as Tunisia and mainland Kenya, are close derivatives of Western European cats, reflecting Western European colonization. The admixed genetics from Western Europe and the Near East cats were subsequently spread to Portuguese colonies in the Americas (Ruiz-Garcia et al. 2005). Although wild felids migrated to the Americas across ancient land bridges and small felids of domestic cat size have been present in South America for millions of years (Johnson et al. 2006; Li et al. 2016), domestic cats only populated the Americas with the arrival of Europeans in the 1500s. This work reinforces domestic felines from the Americas are closely related to those from Europe suggesting an insufficient time for drift or selection to cause genetic distinction (Lipinski et al. 2008).

Cats migrated to Europe and to the east of the Fertile Crescent along with agricultural development and trade (Ottoni et al. 2017; Baca et al. 2018). Pakistan felines tend to have more European influence than other countries in the Middle East, possibly due to the influence and control of the British East India Company in Southern Asia, which is supported by several significant *f*_*3*_ statistics with a contributing population from Europe. Kuwait felines have a higher percentage of Near Eastern ancestry resulting from the location of the country being a center of land and sea trade routes, and a major oil producer resulting in the influx of foreign workers from nearby countries (Shah and Al-Qudsi 1989). India and Sri Lanka both have ancestry admixture from many populations and higher observed heterozygosity attesting to the large amounts of movement of traders due to land and maritime Silk Road routes. Being able to trace these human and cat migration patterns through genetics speaks to the diversity and depth of this sample population reinforcing our ability to narrow the origin of domestication.

European wildcats (*F.s. silvestris)* have many studies focused on the concern of introgression with free-roaming or partially-free-roaming random-bred cats (Beaumont et al. 2001; Witzenberger and Hochkirch 2014; Oliveira et al. 2015; Koch et al. 2016; Mattucci et al. 2019; Quilodrán et al. 2020). A set of 130 European wildcat samples was initially collected as unknown wildcats, and some of these samples were later suggested as hybrids with domestic cat introgression (Oliveira et al. 2015). Hence, the clustering of the 10 wildcat hybrid felines on the periphery of the Western European cats is expected. The 10 wildcat hybrids included in this study have little to no random-bred ancestry in our fastSTRUCTURE analysis and produce no significant *f*_*3*_ statistics, due to the lack of a *F. s. silvestris* reference population. However, the genotypes from these hybrids suggest that European wildcat influence is pervasive throughout populations in Europe but can also be tracked through the genetics of populations in the New World like those in the Americas. Recently, an investigation of cats from China, including a sampling of the Chinese wildcat (*F.s. bieti*), suggested some geneflow between this wild species and domestic cats, but not sufficient to explain the genetic difference between Far Eastern and Western domestic cats. Although a few Asian wildcats (*F.s. ornata*) were included in the Chinese study, the four cats were sampled from one site and specimens from wildcats from the Near East and the Indus Valley were not available (Yu et al. 2021). Thus, further studies are needed to evaluate the complexity of domestication and the influence of admixture with wild populations on modern domestics (Larson and Burger 2013).

Although cats and agricultural species serve very different purposes to humans, the geographic patterns of admixture in cats are a near-perfect reflection of admixture and migration in cattle populations, such as along the Silk Road and in the Americas (Decker et al. 2014). Along with archeological and genetic data, even the cat’s prey, house mice, have also represented bio-proxies for human migration patterns (Rajabi-Maham et al. 2008; Jones et al. 2013; Cucchi et al. 2020; Li et al. 2020).

Overall, worldwide random-bred feline populations exhibit low levels of genetic differentiation even when geographically separated; however, populations on the peripheries of migration can be genetically differentiated. Populations were significantly isolated by distance; among populations, the genetic distance increased as the geographic distance increased. Observed heterozygosity was higher in populations located near the Mediterranean Basin of the Fertile Crescent where archeological evidence points towards the first human-cat interactions. In addition, these populations have a shorter genetic distance to the progenitor species *F.s. lybica*. The origin of domestication for *F.s. catus* is suggested as the coastal regions of the Mediterranean Basin of the Fertile Crescent where cats have high observed heterozygosity and a short genetic distance to the progenitor subspecies. As highly agrarian societies developed, domesticated cats then spread down into the Nile Valley where cultural integration of felines into society slightly decreased heterozygosity and increased the genetic distance from the initial founders. The slightly lower diversity could also be an influence of ancient cultural selections. Mummified Egyptian cats have control region mtDNA mitotypes specific to the mitotype G of contemporary Egyptian cats and a mitotype D highly common in Near and Middle Eastern populations but one mummified cat also had a common mitotype C that has a worldwide distribution (Kurushima et al. 2012), perhaps supported by the Egyptian domestication origin suggested by ancient DNA studies (Ottoni et al. 2017). Further studies on ancient, regional wildcat populations would further decipher cat origins. Cats likely spread throughout Eurasia as agricultural development spread, causing isolation by distance. Once larger sea-bearing vessels facilitated the trade of goods and stores, cat migrations reached more distant ports, including the Americas and Australia in the 1500s. Modern transport of pets has and will continue to increase admixture around the world; however, cat populations in the Americas, Australia, and Madagascar seem to represent the cats of human colonists, where indigenous cats, including wildcats, do not exist. Even the cats of mainland Kenya and the eastern coastal Kenyan islands have genetic signatures similar to Western Europe and the Arabian sea, respectively. While these results are supported by large sample sizes, denser genotypes of these populations would allow for additional methodologies including linkage disequilibrium and haplotype analyses, which could lead to even further clarification of the center of cat domestication.

This study infers the relationships, dispersal, admixture, and genetic distances among worldwide random-bred cats from patterns of genetic polymorphisms, which were unlinked, randomly identified, and assumed to be neutral. Population bottlenecks and effective population sizes cannot be evaluated in the current study. Additional studies including data from various wildcat species/subspecies, particularly *F.s. ornata* from Iraq, Iran, the Indus Valley region, and Northwestern India could further explain the genetic variation seen in cat populations. Genetic and archeological studies from pre-farming cats would be an important addition in further clarifying the cat domestication process. The patterns of genetic diversity and differentiation observed in worldwide random-bred cats parallel those of other species, especially humans once they became farmers, suggesting human history is written in the DNA of domesticated species.

## Supplementary information

Supplementary Tables

Supplementary Figures

Supplementary File 1

Supplementary File 2

## Data Availability

The raw genotypes of individuals are available at FigShare site https://figshare.com/s/7898feb3bb1775405ff9 and 10.6084/m9.figshare.14727177.
