# Supplementary material for: Genetics of randomly bred cats support the cradle of cat domestication being in the Near East
Source: Heredity (Edinb). 2022 Nov 1;129(6):346–55. doi: 10.1038/s41437-022-00568-4 (PMC9708682; doi:10.1038/s41437-022-00568-4)
Supplement: Supplementary file 2 — Supplementary Figures [file 41437_2022_568_MOESM2_ESM.pdf]

Supplementary Figures

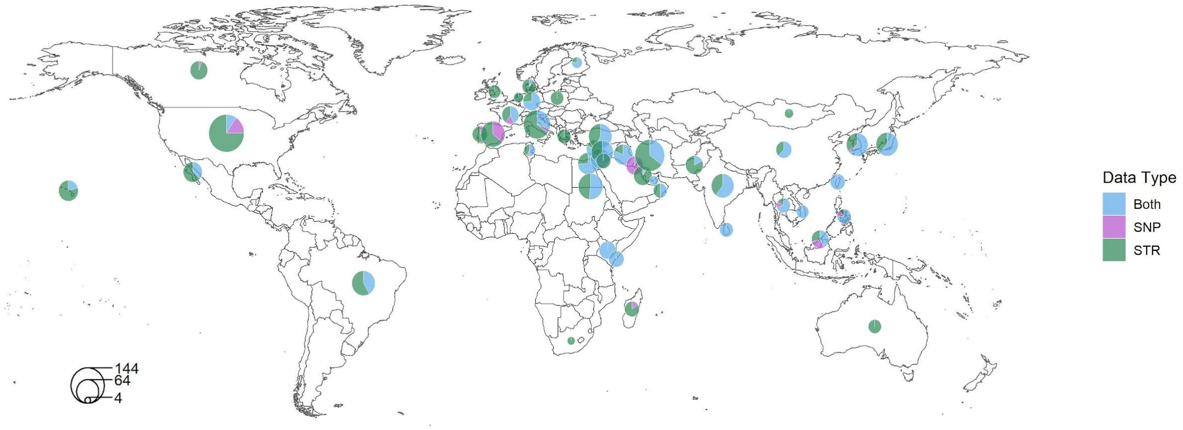

Supplementary Figure 1. Location and data type of sample populations. Each color corresponds to if an individual has both SNP and STR genotypes, only SNP genotypes, or only STR genotypes. The size of the pie is proportional to the number of individuals sampled in that location.

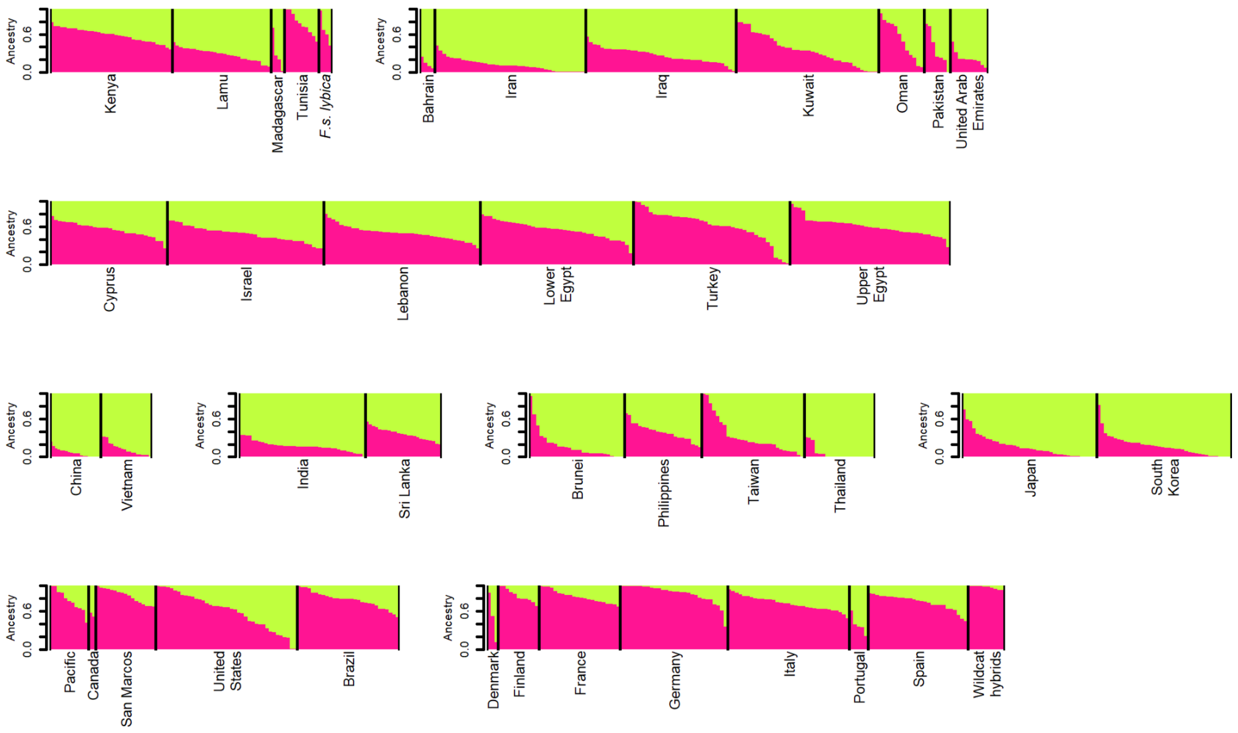

Supplementary Figure 2. SNP fastSTRUCTURE plot of K = 2. Population contributions are represented by different colors, individual vertical bars represent an individual, and populations are separated by black lines.

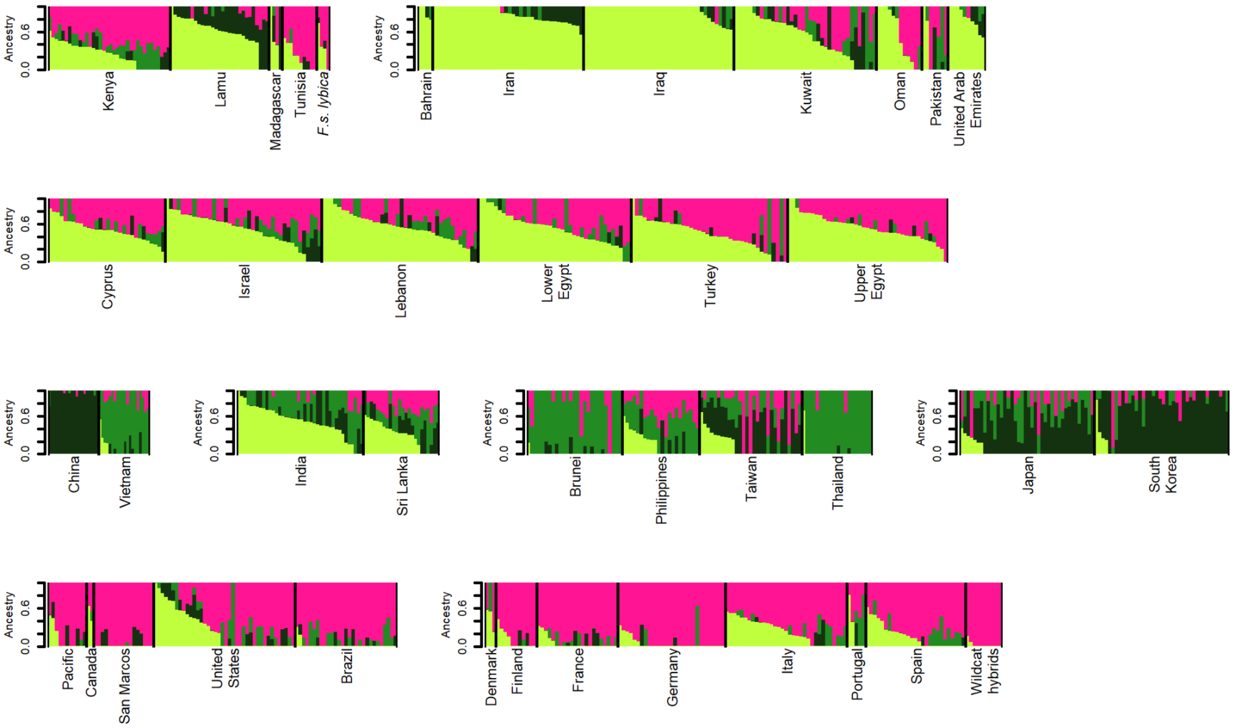

Supplementary Figure 3. SNP fastSTRUCTURE plot of *K* = 4. Population contributions are represented by different colors, individual vertical bars represent an individual, and populations are separated by black lines.

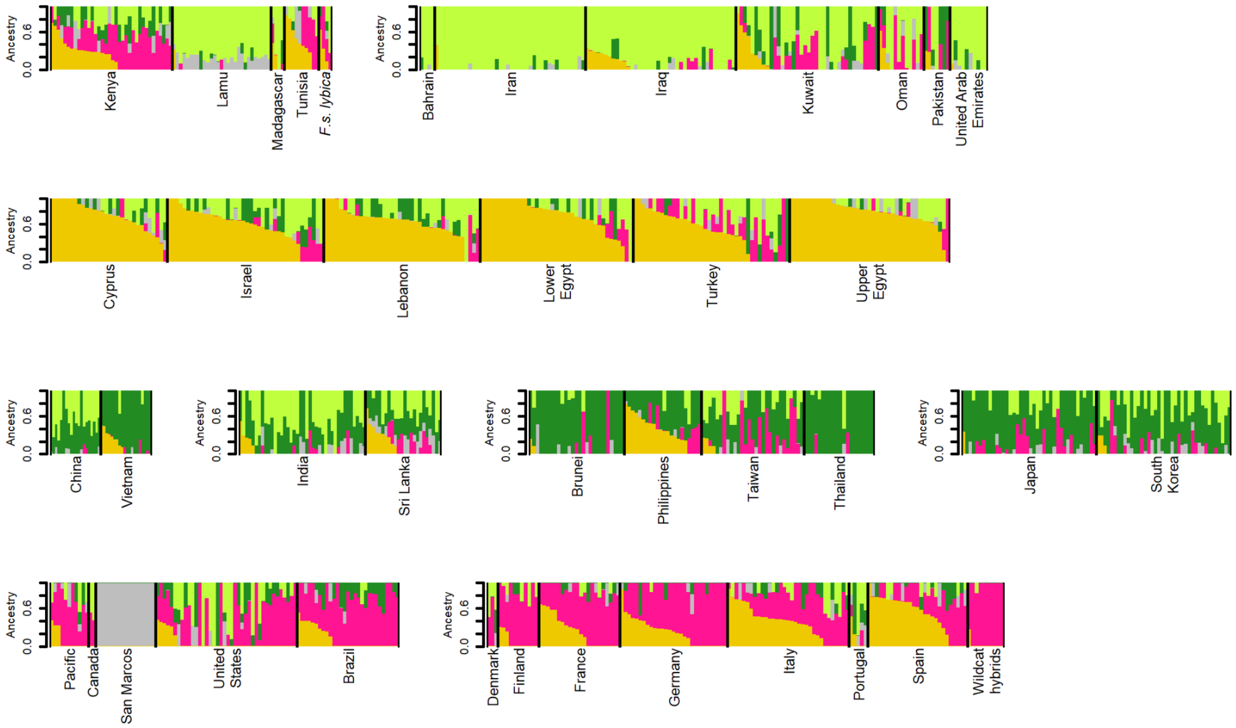

Supplementary Figure 4. SNP fastSTRUCTURE plot of *K* = 5. Population contributions are represented by different colors, individual vertical bars represent an individual, and populations are separated by black lines.

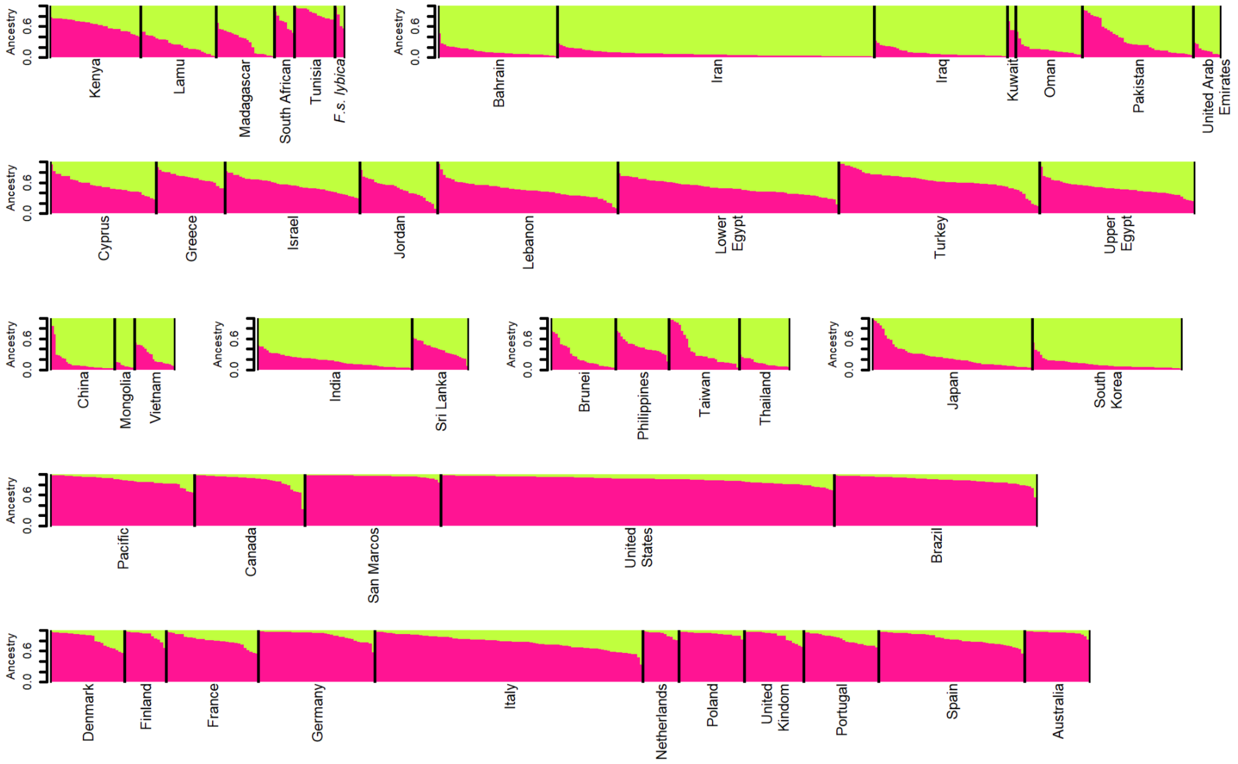

Supplementary Figure 5. STR STRUCTURE plot of *K* = 2. Population contributions are represented by different colors, individual vertical bars represent an individual, and populations are separated by black lines.

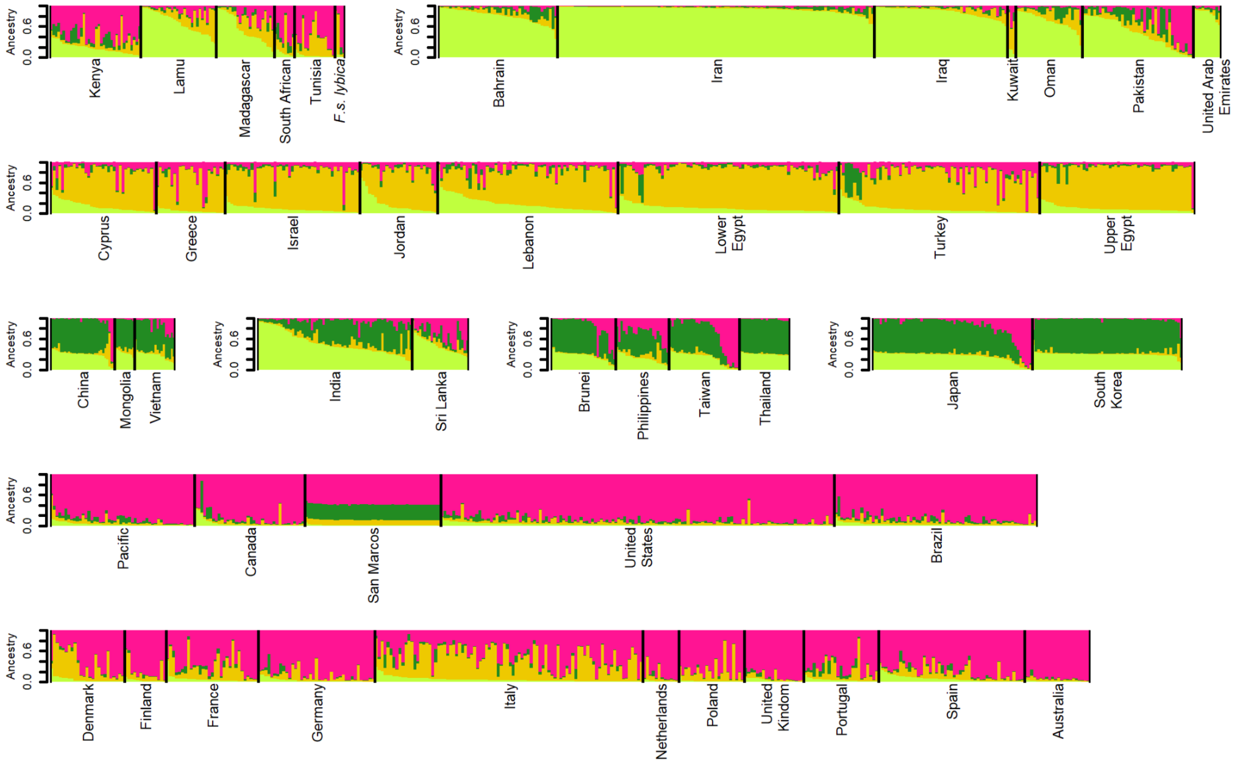

Supplementary Figure 6. STR STRUCTURE plot of *K* = 4. Population contributions are represented by different colors, individual vertical bars represent an individual, and populations are separated by black lines.

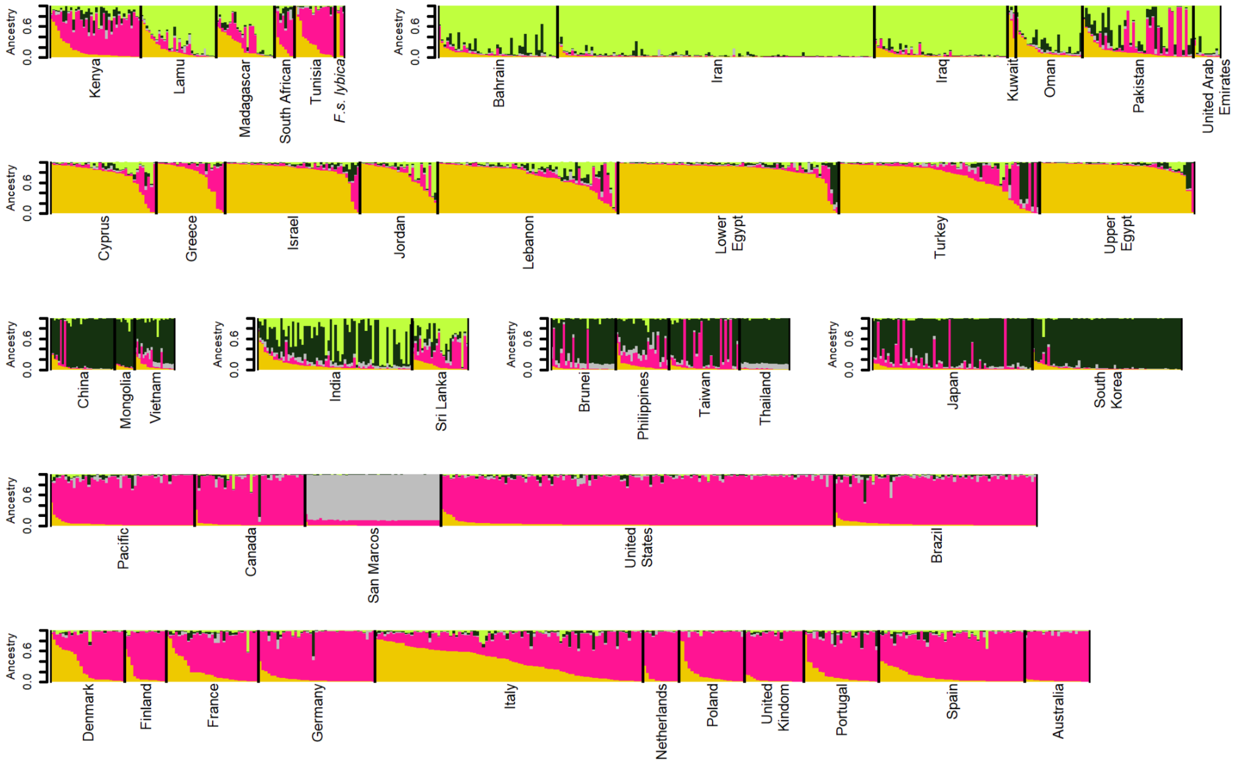

Supplementary Figure 7. STR STRUCTURE plot of *K* = 5. Population contributions are represented by different colors, individual vertical bars represent an individual, and populations are separated by black lines.
